# Altered placental development in undernourished rats: role of maternal glucocorticoids

**DOI:** 10.1186/1477-7827-9-105

**Published:** 2011-08-01

**Authors:** Louiza Belkacemi, Andrea Jelks, Chun-Hung Chen, Michael G Ross, Mina Desai

**Affiliations:** 1 Department of Obstetrics and Gynecology, Los Angeles Biomedical Research Institute at Harbor-UCLA Medical Center, Torrance, California 90502, USA; 2 David-Geffen School of Medicine at UCLA, 10833 Le Conte Ave, Los Angeles, California 90095, USA; 3 Department of Obstetrics and Gynecology, Chang Gung Memorial Hospital-Chia Yi Medical Center, Chia Yi Chia Pu Road (County Way 168), Chia Yi, Taiwan

## Abstract

Maternal undernutrition (MUN) during pregnancy may lead to fetal intrauterine growth restriction (IUGR), which itself predisposes to adult risk of obesity, hypertension, and diabetes. IUGR may stem from insufficient maternal nutrient supply or reduced placental nutrient transfer. In addition, a critical role for maternal stress-induced glucocorticoids (GCs) has been suggested to contribute to both IUGR and the ensuing risk of adult metabolic syndrome. While GC-induced fetal organ defects have been examined, there have been few studies on placental responses to MUN-induced maternal stress. Therefore, we hypothesize that 50% MUN associates with increased maternal GC levels and decreased placental HSD11B. This in turn leads to decreased placental and fetal growth, hence the need to investigate nutrient transporters. We measured maternal serum levels of corticosterone, and the placental basal and labyrinth zone expression of glucocorticoid receptor (NR3C1), 11-hydroxysteroid dehydrogenase B 1 (HSD11B-1) predominantly activates cortisone to cortisol and 11-dehydrocorticosterone (11-DHC) to corticosterone, although can sometimes drive the opposing (inactivating reaction), and HSD11B-2 (only inactivates and converts corticosterone to 11-DHC in rodents) in control and MUN rats at embryonic day 20 (E20). Moreover, we evaluated the expression of nutrient transporters for glucose (SLC2A1, SLC2A3) and amino acids (SLC38A1, 2, and 4). Our results show that MUN dams displayed significantly increased plasma corticosterone levels compared to control dams. Further, a reduction in fetal and placental weights was observed in both the mid-horn and proximal-horn positions. Notably, the placental labyrinth zone, the site of feto-maternal exchange, showed decreased expression of HSD11B1-2 in both horns, and increased HSD11B-1 in proximal-horn placentas, but no change in NR3C1. The reduced placental GCs catabolic capacity was accompanied by downregulation of SLC2A3, SLC38A1, and SLC38A2 expression, and by increased SLC38A4 expression, in labyrinth zones from the mid- and proximal-horns. In marked contrast to the labyrinth zone, the basal zone, which is the site of hormone production, did not show significant changes in any of these enzymes or transporters. These results suggest that dysregulation of the labyrinth zone GC "barrier", and more importantly decreased nutrient supply resulting from downregulation of some of the amino acid system A transporters, may contribute to suboptimal fetal growth under MUN.

## Background

Glucocorticoids (GCs) are critical for fetal organ growth and maturation [1,2], though GCs exposure must occur in a temporally specific pattern. Endogenous (i.e., maternal stress) or exogenous excessive fetal GCs exposure results in reduced fetal growth, and intrauterine growth restricted (IUGR) fetuses have an enhanced susceptibility to hypertension, insulin resistance, and anxiety-related disorders later in life [3-5]. Impairments in fetal growth have been attributed to the direct effects of maternal GCs on the fetus, which prematurely shifts fetal tissue development from a proliferative state to a functionally mature state [6]. Normal fetal growth is dependent on a complex interaction of maternal, placental, and fetal endocrine signals, and GC-mediated fetal growth restriction has also been associated with disturbances in placental growth and function [7,8].

GCs are highly lipophilic, and readily cross placental membranous barrier by passive diffusion. Actions of GCs are mediated via intracellular GC receptor (NR3C1) [9]. During pregnancy high levels of cortisol (human) [10] and corticosterone (rat) [11] are prevalent within the maternal circulation. However, maternal GCs are largely excluded from the fetus [12]. The difference in GC concentrations between the maternal and fetal plasma is attributed to the special transport and permeability properties of the placenta [13]. Specifically, the placenta protects the fetus against maternal cortisol or corticosterone by 11-hydroxysteroid dehydrogenase (HSD11B)-mediated enzymatic oxidation of these hormones to their biological inactive forms. To date, two HSD11B isoforms have been cloned. HSD11B type 1 (HSD11B-1) isoform possesses both oxidase and reductase activities but functions mainly as a reductase, converting cortisone and 11-dehydrocorticosterone (11-DHC) to cortisol, and corticosterone, respectively. Conversely, HSD11B type 2 (HSD11B-2) acts as an oxidase that inactivates bioactive cortisol and corticosterone to inactive cortisone and 11B-hydrocorticosterone. Thus, HSD11B-2 constitutes a placental GC "barrier" that could contribute to the modulation of fetal growth. Although the activity and expression of HSD11B-2 within the placenta correlates with birth weight [14,15] and HSD11B-2 gene expression is reduced in IUGR rats and human gestations [14,16], relative changes in HSD11B-1/2 activity/expression cannot fully characterize the amount of active/inactive GCs within the placenta as there is an influx of GCs from maternal [17] and fetal [18] compartments to the placenta during pregnancy.

Maternal global undernutrition (MUN), or selective protein deprivation during pregnancy increase maternal GCs plasma levels, and reduce fetal and placental weights [19-21]. Consistent with this, we have shown that 50% MUN during the second period of pregnancy (E10-E20) results in reduced placental and fetal weights at term gestation in rat. We hypothesize that MUN during pregnancy associates with increased maternal GCs levels, and decreased placental HSD11B enzyme as well reduced placental nutrient transporters with subsequent negative effects on placental and fetal growth. Our first objective was therefore to determine the impact of MUN on maternal GC levels, and on the placental expression of HSD11B-1/2, and NR3C1 in the basal zone (site of hormone production) and in the labyrinth zone (site of maternal-fetal nutrient exchange) from both mid- and proximal-horn placentas at E20. We focused on fetuses and placentas from mid- and proximal-horn positions to evaluate any differential impact by MUN. The mid-horn (site that receives the lowest level of maternal blood supply) and proximal-horn (site that receives the highest level of maternal blood supply) positions were specifically investigated. Glucose transporters are critical for maintaining the supply of glucose, the primary substrate for fetal oxidative metabolism, and sodium-dependent amino acid transport system A is essential for supplying neutral amino acids to the fetus [22-24], and both types of transporters are affected by intracellular levels of GCs in many cell types, including placental trophoblast cells [25]. Consequently, our second objective was to assess the placental changes in the expression of glucose transporter 1 (SLC2A1, previously called GLUT1) and glucose transporter 3 (SLC2A3, previously called GLUT3), together with the three known system A amino acid transporters SLC38A1, 2, and 4 (previously called SNAT1, 2, and 3) [26] in basal and labyrinth zones from mid- and proximal-horn placentas in response to MUN.

## Methods

### Animals

Studies were approved by the Animal Research Committee of the Los Angeles BioMedical Research Institute at Harbor-UCLA (LABioMed), and were in accordance with the American Association for Accreditation of Laboratory Care (AALC), and National Institutes of Health (NIH) guidelines. Eight-week-old first-time-pregnant Sprague Dawley rats (230-240 g body weight) (Charles River Laboratories Inc., CA) were housed in a facility with constant temperature and humidity, a controlled 12 h light/dark cycle, and an ad libitum diet (AdLib) of standard laboratory chow (protein 23%, fat 4.5%, metabolizable energy 3030 kcal/kg; Lab Diet 5001, MO). At embryonic age 10 (E10) based upon day of expelled plug, dams were randomly allocated to a control diet (N = 6) in which they were continued on the AdLib diet or a 50% food-restricted (MUN) diet (N = 6) that was determined by quantifying normal intake of the rats that were fed AdLib at the equivalent stage of gestation. Respective diets were continued throughout the remainder of gestation. At E20, dams were weighed before sacrifice.

### Maternal blood collection and plasma corticosterone quantification

Maternal blood obtained by cardiac puncture from AdLib (N = 6) and MUN (N = 6) dams was collected into heparinized tubes, centrifuged at 9,000 g for 10 minutes at 4°C, and plasma was frozen at -80°C until use. Maternal plasma corticosterone levels were determined by radioimmunoassay (Diagnostic Systems Laboratories Inc., TX) using a highly specific corticosterone antiserum, as per manufacturer's instructions. The detection threshold was 2.7 ng/ml. The intra- and inter-assay variations were 2.4 and 4.4%, respectively.

### Fetal and placental tissue collections

AdLib (N = 6) and MUN (N = 6) dams were sacrificed E20 using an overdose of 4% isoflurane. The uterus was delivered through a mid-line incision, and the gestational sacs were removed. Fetuses and placentas were removed and labeled according to position in each uterine horn. Excess fluid was blotted from the fetuses and their wet weights obtained using an electronic scale with an accuracy of ± 0.1 mg (Mettler Instrument Corp, Model AE50, Hightstown, NJ). Proximal-horn gestations were those closest to the cervix; mid-horn gestations were in the middle of the uterine horn. When the number of fetuses was odd, there was one mid-horn gestation, whereas when the number of fetuses was even, there were two mid-horn gestations. We separated placentas from mid and proximal-horn positions to evaluate uterine position (difference in maternal blood supply) in relation to AdLib or MUN diets. The placentas were dissected into basal and labyrinth zones, the two morphologically and functionally distinct zones. Only uterine horns with five to seven fetuses were used. Placentas were trimmed of membranes and weights recorded. The two zones were then individually weighed and flash-frozen in liquid nitrogen for protein extraction.

### Protein extraction, SDS-PAGE, and Western blot analysis

Placentas were separated into basal and labyrinth zones, and sonicated on ice in the T-PER tissue protein extraction reagent buffer (Thermo Scientific, IL) that contained protease inhibitors (HALT cocktail, Thermo Scientific). Protein concentration was determined by bicinchoninic acid (BCA) solution (Thermo Scientific). All protein fractions were frozen at -80°C until use.

The effect of MUN on placental expression of HSD11B-1, HSD11B-2, and NR3C1, and SLC2A1, SLC2A3, SLC38A1, SLC38A2 and SLC38A4 protein levels was evaluated by Western blot analysis of the tissue lysates. For each sample, 50 μg of protein were separated on a 12% polyacrylamide gel (Invitrogen Corp., CA). A positive control (an aliquot of a cell lysate with the corresponding antigen overexpressed) was also loaded with each immunoblot (Table 1). The separated proteins were transferred electrophoretically to Immobilon-P membranes (BioRad Laboratories, CA). Membranes were incubated in a blocking solution (5% powdered non-fat milk in Tris buffer saline [TBS] and 0.01% Tween-20 [TBS-T]) at room temperature for 1 h, and then in the same solution containing a primary antibody that reacts with HSD11B-1, HSD11B-2, NR3C1, or one of the nutrient transporters SLC2A1, SLC2A3, SLC38A1, SLC38A2, and SLC38A4 (see Table 1 for dilutions), at 4°C overnight. After washes in TBS-T, membranes were incubated with the corresponding horseradish peroxidase secondary antibody (Table 1) at room temperature for 1 h followed by washes in TBS-T. Proteins were visualized by the enhanced chemiluminescence signaling system (GE Healthcare, NJ) after which blots were exposed to autoradiography film. The resulting bands were compared by scanning densitometry. To ensure equal loading, protein blots were stripped and reprobed with glyceraldehyde-3-phosphate dehydrogenase (GAPDH) (1:1000; Millipore Corp., MA). Placental GAPDH expression has been previously demonstrated to remain stable under conditions of MUN [27].

**Table 1:** Antibodies used in Western blot analysis

Primary antibodies and their commercial source	First antibody dilution	Horse radish peroxidase-linked secondary antibodies, and their commercial source	Positive controls	Secondary antibody dilution
*Rabbit polyclonal* *HSD11B-1 (Cayman Ann Arbor, MI)*	1:500	Rabbit immunoglobulin G (Millipore,	Rat liver tissue lysate	1:2500
*Sheep polyclonal* *HSD11B-2 (Chemicon*, *Temecula, CA)*	1:4000	Sheep immunoglobulin G (Upstate, Temecula, CA)	Rat liver tissue lysate	1:5000
*Rabbit polyclonal* *GR (Abcam, Cambridge, MA)*	1:3500	Rabbit immunoglobulin G (Millipore, MA)	Rat liver tissue lysate	1:2500
*Rabbit polyclonal* *SLA2C1 (Santa Cruz, CA)*	1:700	Rabbit immunoglobulin G (Millipore, MA)	H4 cell lysate (Santa Cruz)	1:2500
*Rabbit polyclonal* *SLA2C3 (Millipore Corp., CA)*	1:700	Rabbit immunoglobulin G (Millipore Corp., Ca)	SLAC2A3 peptide (Millipore)	1:2500
*Rabbit polyclonal* *SLC38A1 (Santa Cruz, CA)*	1:700	Rabbit immunoglobulin G (Millipore Corp., MA)	293T cell lysates (Santa Cruz)	1:2500
*Rabbit polyclonal* *SLC38A2 (Santa Cruz, CA)*	1:700	Rabbit immunoglobulin G (Millipore Corp., MA)	U-87MG cell lysates (Santa Cruz	1:2000
*Rabbit polyclonal* *SLC38A4 (Santa Cruz, CA)*	1:700	Rabbit immunoglobulin G (Millipore Corp., MA)	293 T cell lysate (Santa Cruz)	1:2000

### Statistical Analysis

Maternal corticosterone levels, and maternal, fetal, and placental weights, as well as Western blots were analyzed using NCSS97 software (NCSS, UT). Maternal corticosterone levels were compared using a two-tailed Student t-test. Maternal weights were compared using a one-way analysis of variance (ANOVA). Fetal weights and placental protein expression levels were compared using a two-way ANOVA (with horn positions, and diet as sources of variation) followed by Tukey-Kramer post hoc test. Further, placental weights were compared with a three-way ANOVA (with horn positions, zones, and diet as causes of variation). When interaction occurs between these factors, subsequent analyses were carried out using a one-way ANOVA test. Protein expression in the labyrinth zone were compared using a two-way ANOVA (with diet and positions are causes of variation) followed by Tukey-Kramer post hoc test. Since differences between zones and placental positions were evident, data are presented separately for basal and labyrinth zones, and mid- and proximal-horn placentas. Correlations between changes in means of maternal GC levels and fetal or placental weights at term gestation were performed using Pearson's correlation coefficient. Values are expressed as means ± SE and considered significant at P < 0.05.

## Results

### Maternal corticosterone levels

**Figure 1:**
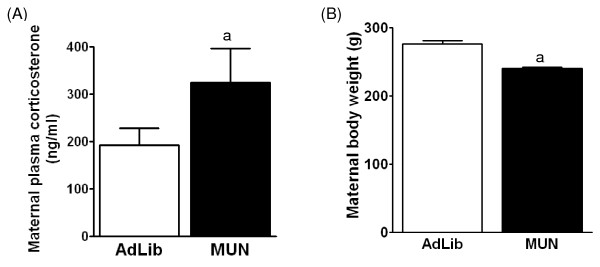
**Maternal corticosterone levels and weight in MUN and AdLib dams at E20 gestation**. Maternal serum corticosterone levels (B) in MUN (black bars) and AdLib (open bars) dams at E20. ^a ^indicates a significant difference between zone weights from MUN vs. AdLib.

Maternal plasma corticosterone levels were significantly higher in MUN dams at E20 compared to AdLib dams (Figure 1A; P < 0.01, Student t-test), consistent with a maternal stress response.

### Maternal weight

Prior to the initiation of MUN (E10), there was no difference in body weight between the MUN and AdLib dams (data not shown). At E20, the MUN dams had significantly lower body weights compared to those from AdLib dams (Figure 1B; P < 0.001, one-way ANOVA).

### Fetal weight

**Figure 2:**
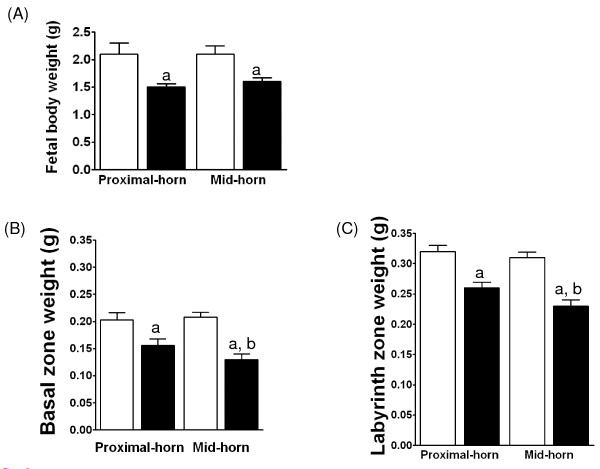
**Fetal and placental weights**. Fetal body weights from MUN and AdLib mid- and proximal-horns (A). Placental basal (C) and labyrinth (D) weights from MUN (black bars) and AdLib (open bars) mid- and proximal-horns. ^a ^indicates a significant difference between zone weights from MUN vs. AdLib. ^b^indicates a significant difference between proximal- and mid-horn placental positions.

MUN E20 fetuses from either mid-horn or proximal-horn placentas weighed significantly less than position-matched fetuses from AdLib controls (Figure 2A; P < 0.02, two-way ANOVA). In contrast, MUN and AdLib fetal weights from mid-horn positions were comparable to the respective fetal weights from proximal-horn positions (Figure 2A; one-way ANOVA).

### Placental zone weights

MUN basal (Figure 2B) and labyrinth (Figure 2C) zones from either mid-horn or proximal-horn positions weighed significantly less than AdLib zone- and position-matched placentas (P < 0.05, three-way ANOVA). Among MUN placentas, the basal and the labyrinth zones from mid-horn placentas weighed significantly less than zone-matched tissues from proximal-horn sites (Figure 2B-C; P < 0.05, one-way ANOVA), while uterine position had no impact on the weights of the two placental zones in AdLib gestations at E20.

### Correlation studies

We assessed the correlation between maternal corticosterone levels with fetal or placental weights in MUN rats. We found a modest inverse correlation between maternal corticosterone levels with fetal weight (fetal: r = -0.22), mid-horn placentas (r = -0.36), or proximal-horn placentas (r = -0.31), though without any statistical significance.

### Placental HSD11B-1 and -2, and NR3C1 protein expression

**Figure 3:**
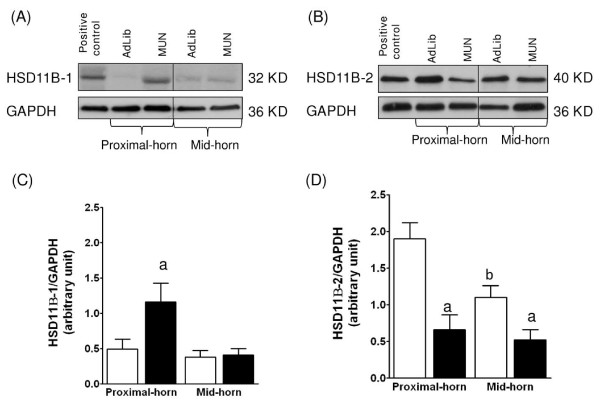
**Effects of MUN on the level of HSD11B-1 and HSD11B-2 protein expression in labyrinth zone from mid- and proximal-horn placentas**. Representative immunoblots of HSD11B-1 (A) and HSD11B-2 (B) protein expression. Densitometric analysis of HSD11B-1 (C) and HSD11B-2 (D) protein expression. ^a ^indicates a significant difference between zone weights from MUN (black bars) vs. AdLib (open bars). ^b^indicates a significant difference between proximal- and mid-horn placental positions.

For each of the protein, we detected a single immunoreactive band of the expected molecular weight on Western blots of placental protein lysates (Figure 3A-B). In the labyrinth zone, the expression of HSD11B-1 (GC activation) was significantly increased MUN placentas from proximal-horn compared to position matched-AdLib controls (Figure 3C; P < 0.05, two-way ANOVA) and remained statistically significant upon Tukey-Kramer post hoc analysis, whereas HSD11B-2 (GC inactivation) was significantly lower in MUN labyrinth tissue from both horns as compared to the AdLib placentas (Figure 3D; P < 0.05, two-way ANOVA). NR3C1 protein was similarly expressed in MUN and AdLib specimens from either mid- or proximal horns (data not shown). When comparing positions, MUN placental labyrinth zones from mid-horn placentas did not show any significant difference for either HSD11B-1 or HSD11B-2, compared to the respective proximal-horn placentas using one-way ANOVA. In contrast, in the AdLib, HSD11B-2 expression was significantly decreased in the labyrinth zone of mid-horn placentas, compared to the zone-matched proximal-horn placentas (Figure 3D; P < 0.05, one-way ANOVA).

In the basal zone, expression levels HSD11B-1, HSD11B-2, and NR3C1 proteins from either mid-horn or proximal-horn positions were unchanged in MUN and compared to AdLib controls (results not shown).

### Placental glucose and amino acid transporters expression

**Figure 4:**
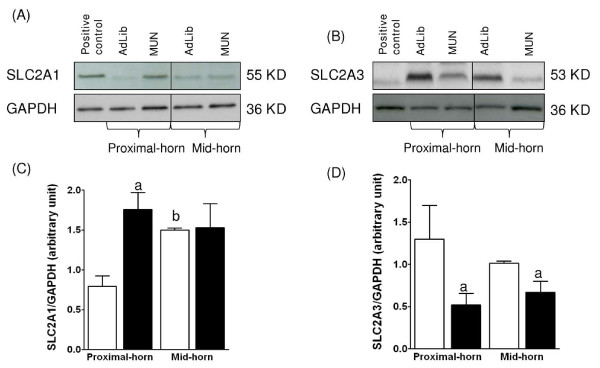
**Effects of MUN on the level of SLC2A1 and SLC2A3 protein expression in labyrinth zone from mid- and proximal-horn placentas**. Representative immunoblots of SLC2A1 (A) and SLC2A3 (B) protein expression. Densitometric analysis of SLC2A1 (C) and SLC2A3 (D) protein expression. ^a^indicates a significant difference between zone weights from MUN (black bars) vs. AdLib (open bars). ^b^indicates a significant difference between proximal- and mid-horn placental positions.

**Figure 5:**
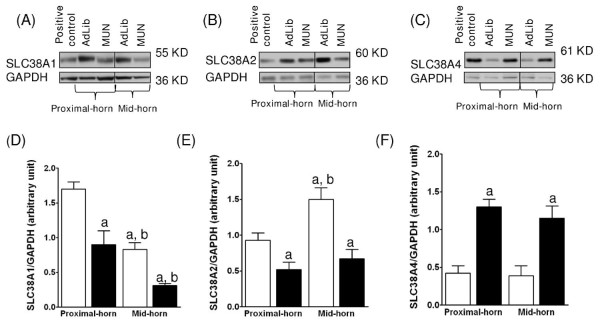
**Effects of MUN on the level of SLC38A protein expression in labyrinth zone from mid- and proximal-horn placentas**. Representative immunoblots of SLC38A1 (A), SLC38A2 (B) and SLC38A 4 (C) protein expression. Densitometric analysis of SLC38A1 (D), SLC38A2 (E), and SLC38A4 (F) protein expression. ^a ^indicates a significant difference between zone weights from MUN (black bars) vs. AdLib (open bars). ^b^indicates a significant difference between mid- and proximal-horn placental positions.

As GCs affect both placental glucose [22] and amino acid [28] transporter systems, we determined the expression levels of facilitative glucose transporters SLC2A1 and SLC2A3, and neutral amino acids transporters SLC38A1, SLC38A2 and SLC38A4 proteins. For each of these proteins, we detected a single immunoreactive band of the expected molecular weight on Western blots of placental protein lysates (Figures 4A-B, 5A-C). In the labyrinth zone, SLC2A1 expression was significantly upregulated in the proximal-horn (Figure 4C; P < 0.04, two-way ANOVA) but unchanged in mid-horn placentas from MUN dams, compared to the respective AdLib zones from mid-horn or proximal-horn placentas. In the basal zone, SLC2A1 was unchanged in either mid-horn, or proximal-horn placentas of MUN dams compared to the respective AdLib zones from mid-horn or proximal-horn placentas (results not shown). Since previous studies have shown that SLC2A3 is not expressed in the basal zone in rat [29], we quantified SLC2A3 expression in the labyrinth zone only. SLC2A3 was significantly downregulated in the labyrinth zones from either MUN mid- or proximal-horn compared to the respective AdLib zones from mid-horn or proximal-horn placentas (Figure 4D; P < 0.05, two-ANOVA).

When comparing positions, MUN placental labyrinth zones from mid-horn placentas did not show any significant difference for either SLC2A1 or SLC2A3, compared to the respective proximal-horn placentas. Conversely, in the AdLib placentas, SLC2A1 expression was significantly increased in the labyrinth zone from mid-horns, compared to the proximal-horn positions (Figure 4C; P < 0.05, one-way ANOVA).

Similarly to SLC2A3, SLC38A1 and SLC38A2 proteins were significantly decreased in the MUN labyrinth zone, though not in the MUN basal zone, of either mid-horn or proximal-horn placentas compared to the respective AdLib zones from mid-horn or proximal-horn placentas (Figure 5D-E; P < 0.05, two-way ANOVA). Conversely, SLC38A4 was significantly increased in the MUN labyrinth zone, of either mid-horn or proximal-horn placental positions compared to AdLib zones from mid-horn or proximal-horn placentas (Figure 5F; P < 0.05, two-way ANOVA) and remained statistically significant upon Tukey-Kramer post hoc analysis. MUN basal zone expression of SLC38A4 was unchanged at either uterine position.

When comparing positions, MUN placental labyrinth zones from mid-horn placentas exhibited significantly lower SLC38A1 protein expression, compared to the labyrinth zone of proximal-horn placentas (Figure 5D; P < 0.05, one-way ANOVA). Similarly, in AdLib placentas, SLC38A1 expression levels were significantly lower in the labyrinth zone of mid-horn compared to zone-matched proximal-horn placental positions (Figure 5D-E; P < 0.05, One-way ANOVA). Moreover, in MUN placentas, labyrinth zones from mid-horn placentas exhibited significantly lower SLC38A2 protein expression, compared to the labyrinth zones of proximal-horn placentas. Similarly, SLC38A2 expression levels in AdLib placentas were significantly lower in mid-horn compared to zone-matched proximal-horn placental positions (Figure 5D-E; P < 0.05, One-way ANOVA). Notably, SLC38A4 expression levels in either MUN or AdLib placental zones were unchanged in mid- vs. proximal-horn placental positions (Figure 5F).

## Discussion

This study addressed the impact of an MUN-mediated maternal stress and nutrient response on fetal and placental development in rats between E10-E20. Maternal food restriction produced a predictable stress response in the mother, as evidenced by elevated maternal plasma corticosterone, and a reduction in fetal weight and hypotrophy of the basal and labyrinth zones of the placenta. The reduction in fetal and placental weights was observed in both mid- and proximal-horn positions, the uterine regions which receive the least and greatest levels of maternal blood flow, respectively. The most significant changes in placental tissue occurred in the placental labyrinth zone. Labyrinth tissue, which mediates feto-maternal nutrient exchange, exhibited decreased expression of HSD11B-2, the enzyme that oxidizes and deactivates corticosterone to 11-dehydrocorticosterone (cortisol to cortisone in humans). In addition, MUN placentas expressed increased HSD11B-1, the reductase that produces corticosterone (cortisol in humans), in proximal-horn placentas. There was no change in NR3C1 expression at either uterine position. Nutrient transporters, including SLC38A1 and SLC38A2 were significantly downregulated in MUN placental labyrinth zone from either mid- or proximal-horns. Conversely, SLC38A4 was upregulated in the labyrinth zone from both placental positions. In marked contrast to the labyrinth zone, the basal zone did not show changes in any of these metabolizing enzymes or transporters. Collectively, our results suggest that the increase in maternal GCs following MUN-mediated stress is associated with both dysregulation of the placental GC barrier and the decrease in overall maternal nutrients availability to the fetus leading to suboptimal fetal growth. Although, placental transport of selective nutrient to the fetus may still be increased if SLC2A3 and SLC38A4 were the major contributors, but this did not prevent fetal weight loss in the MUN gestations.

Higher levels of maternal GCs and downregulation of HSD11B-2, may disrupt several essential developmental processes [6], including placenta through increased local GC levels that will affect placental weight and development. Thus, it is possible that the compromised placental weight we observed under MUN is mediated, in part, by the HSD11B-2 downregulation, and the resulting oversupply of corticosterone to the placenta. In support of this, HSD11B-2 knockout mice (HSD11B-2-/-) result in substantially smaller placentas than congenic littermate controls (HSD11B-2+/+) from HSD11B-2+/- crosses [4]. Although the loss of the HSD11B-2 enzyme may be sufficient to cause placental hypotrophy without a direct maternal contribution, increased maternal GCs may exacerbate these effects on the placenta. However, in our study we found only a modest inverse correlation between maternal corticosterone levels, and MUN placental weights, suggesting a rather limited effect of increased maternal GCs on placental growth. It is therefore possible that any increase in placental corticosterone following MUN insult may result, at least partially, from the downregulation of HSD11B-2 expression/activity, and or overexpression/ hyperactivity of HSD11B-1. Although, additional experiments are required to differentiate the contribution of the two HSD11B isoforms on placental weight and function.

Contrary to our findings, Lesage et al. [30] found that normalization of maternal corticosterone levels by adrenalectomy plus corticosterone supplementation in a rat MUN model with reduced HSD11B-2 expression did not affect placental growth. The *discrepancies *in these observations may be due to differences in methodology, as we specifically controlled for placental location (mid- and proximal-horns) whereas Lesage's study did not control for placental position. Alternatively, the growth restriction in the MUN placentas may rather stem from lack of maternal nutrients, or some other MUN-activated signaling pathways. Consistent with this, rat dams fed low protein diet (an insult that has similarities with MUN), or fed 50% MUN (identical to our study) have increased corticosterone levels [19-21], and decreased placental weight as well as placental HSD11B-2 expression [19,20]. Interestingly, in sheep, elevated maternal plasma cortisol concentrations following 10 days MUN in late gestation did not persist when the duration of MUN was extended to 20 days nor did it affect placental weight, suggesting an adaptation to a new plan of nutrition over time [21]. It is worth noting that these studies and our's did not discriminate between the contribution of increased maternal GCs and MUN to the observed experimental endpoints, and both parameters may alter placental development either independently or in concert. Hence, the signaling mechanisms leading to HSD11B-2 underexpression and in some regions HSD11B-1 overexpression and subsequent placental growth restriction in the MUN pregnancies warrant further study.

As reduced placental MUN labyrinth zone weight suggested an impaired placental nutrient transport capacity, we examined the expression of glucose and amino acid transporters. SLC2A1 and SLC2A3 glucose transporter proteins were divergently regulated in the labyrinth zone of MUN rats. The upregulation of SLC2A1 in the labyrinth zone from the proximal-horn placenta implies an increase in glucose transport capacity that may be a compensatory mechanism to raise fetal glucose supply, as SLC2A1, which is normally localized at the site of entry and exit in the placental barrier, facilitates materno-fetal glucose transport [31]. Conversely, the unchanged SLC2A1 expression in the labyrinth zone from the mid-horn may not be influenced by MUN. Reduced SLC2A3 expression in MUN placentas was reported previously [30], and our findings are consistent with their results. Thus, it is possible that under MUN conditions, SLC2A3, which has a lower Km (*1.8 mM) *for glucose compared to SLC2A1 Km *(2-5 mM*) [32-34] may be more sensitive to intracellular variations of glucose concentrations, compared to SLC2A1. Alternatively, the decrease in SLC2A3 in the MUN placentas could be a protective measure to limit glucose transport out of the placenta [30]. Since the fetus is almost entirely dependent on maternal glucose passing through the placenta, inadequate transplacental passage of glucose for an extended period of time will likely affect fetal growth and development. The decrease in the expression of the amino acid transporters SLC38A1 and SLC38A2 in the labyrinth zone may further impair fetal growth. Unlike SLC38A1/2, SLC38A4 expression was upregulated in the labyrinth zone from both horns, which may again reflect a compensatory mechanism aimed at limiting amino acid clearance [15]. Whether transporters dysregulation in the MUN placentas is solely due to MUN or oversupply of corticosterone following MUN, or both remains to be investigated.

Analysis of transporter expression by position within the horns showed significantly lower SLC38A1 protein expression in MUN mid-horn placentas compared to proximal-horn placentas, possibly due to differences in maternal blood supply [34] or levels of local corticosterone or both. This difference may also reflect a more pronounced dysfunction of certain protein from system A in the mid-horn position. Conversely, SLC2A1 and SLC38A2 expression upregulation in placental AdLib labyrinth zone from mid- compared to proximal-horn positions, may represent a compensatory mechanism. Whereas lower SLC38A1 expression mid-horn placentas compared to proximal-horn placentas may associate with differences in maternal blood supply in the AdLib. Overall, our results demonstrate zone-specific and certain position-specific changes in amino acid transporter expression in MUN placentas, resulting in impaired placental growth and most likely in placental dysfunction. The positional difference in the AdLib placentas may suggest the existence of compensatory mechanisms.

Maternal plasma corticosterone levels were negatively correlated with MUN fetal weights at term gestation but without significance, implying that MUN fetal weight reduction may not result from a direct increase in maternal corticosterone. In support of our findings Lesage et al. [30] study demonstrated that MUN induced IUGR similarly in both normal and in pre-adrenalectomized MUN rats supplemented with control levels of corticosterone. These investigators also reported elevated plasma corticosterone in rat fetuses from MUN gestations, as well as decreased fetal adrenal weight and decreased adrenocorticotropic hormone (ACTH) levels, suggesting suppression of intrinsic fetal adrenal corticosterone production [21]. Their findings indicate that perhaps the fetal growth restriction in the MUN fetus was not associated with a direct increase in GCs. Based on these findings; we speculate that decreased nutrient transport following downregulation of SLC38A1/2, may be the main contributor to the fetal growth restriction in the MUN pregnancies. In support of our data Jansson et al. [35] showed that decrease placental amino acid transport precedes IUGR, and hence is probably a contributing cause rather than just a secondary consequence of it.

**Figure 6:**
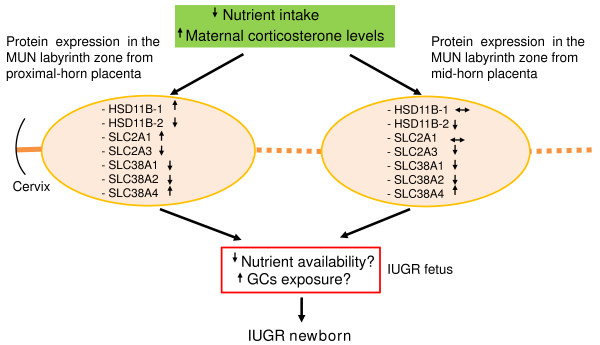
**Increased maternal corticosterone levels and altered placental labyrinth zone expression of HSD11B enzymes and nutrient transporters in undernourished rat gestations at E20**.

In summary, suboptimal maternal nutrition enhances maternal GCs production, reduces placental weight and placental expression of HSD11B-2 as well as nutrient transporters, all of which may contribute to compromised fetal growth (Figure 6). Because maternal GCs modestly correlated with decreased fetal weight in the MUN fetuses, we speculate that the MUN-decreased fetal growth could be related, mainly, to disruption of placental transfer of nutrients and/or disturbance of their use by fetal tissues (Figure 6). However, this study does not rule out the possibility that maternal GCs may participate indirectly in programming processes that contribute to fetal suboptimal growth. The insult on the mid-horn placentas did not translate into more severe effect on the corresponding fetus as there was no significant difference between MUN fetuses from mid-horn vs. proximal-horn positions, which may indicate a compensatory mechanism. Subsequent reduction in newborn weights associates with a higher incidence of metabolic syndromes in adulthood [34]. Remarkably, hypertension caused by maternal dietary protein restriction can be prevented by pharmacological blockade of GC biosynthesis in pregnant rats and offsprings [35], implicating GC inhibitors as a therapeutic tool to improve placental and birth defects associated with undernutrition during pregnancy. In our model, since maternal GCs may have limited direct impact on fetal growth, improved maternal dietary intake alone may prevent or ameliorate suboptimal placental and fetal growth in MUN gestations.

## References

[B1] McNeil CJ Nwagwu MO Finch AM Page KR Thain A McArdle HJ Ashworth CJ Glucocorticoid exposure and tissue gene expression of 11beta HSD-1, 11beta HSD-2, and glucocorticoid receptor in a porcine model of differential fetal growth Reproduction 2007 133 653 661 10.1530/rep.1.01198 17379659

[B2] Ni XT Duan T Yang Z Guo CM Li JN Sun K Role of human chorionic gonadotropin in maintaining 11beta-hydroxysteroid dehydrogenase type 2 expression in human placental syncytiotrophoblasts Placenta 2009 30 1023 1028 10.1016/j.placenta.2009.10.005 19880179

[B3] Benediktsson R Lindsay RS Noble J Seckl JR Edwards CR Glucocorticoid exposure in utero: new model for adult hypertension Lancet 1993 341 339 341 10.1016/0140-6736(93)90138-7 8094115

[B4] Holmes MC Abrahamsen CT French KL Paterson JM Mullins JJ Seckl JR The mother or the fetus? 11beta-hydroxysteroid dehydrogenase type 2 null mice provide evidence for direct fetal programming of behavior by endogenous glucocorticoids The Journal of neuroscience: the official journal of the Society for Neuroscience 2006 26 3840 3844 10.1523/JNEUROSCI.4464-05.2006 PMC6445356 16597738

[B5] Nyirenda MJ Lindsay RS Kenyon CJ Burchell A Seckl JR Glucocorticoid exposure in late gestation permanently programs rat hepatic phosphoenolpyruvate carboxykinase and glucocorticoid receptor expression and causes glucose intolerance in adult offspring The Journal of clinical investigation 1998 101 2174 2181 10.1172/JCI1567 9593773 PMC508805

[B6] Seckl JR Holmes MC Mechanisms of disease: glucocorticoids, their placental metabolism and fetal 'programming' of adult pathophysiology Nature clinical practice Endocrinology & metabolism 2007 3 479 488 17515892 10.1038/ncpendmet0515

[B7] Hewitt DP Mark PJ Waddell BJ Glucocorticoids prevent the normal increase in placental vascular endothelial growth factor expression and placental vascularity during late pregnancy in the rat Endocrinology 2006 147 5568 5574 10.1210/en.2006-0825 16959835

[B8] Mairesse J Lesage J Breton C Breant B Hahn T Darnaudery M Dickson SL Seckl J Blondeau B Vieau D Maternal stress alters endocrine function of the feto-placental unit in rats American journal of physiology Endocrinology and metabolism 2007 292 E1526 1533 10.1152/ajpendo.00574.2006 17264224

[B9] Kino T Chrousos GP Glucocorticoid and mineralocorticoid receptors and associated diseases Essays in biochemistry 2004 40 137 155 15242344 10.1042/bse0400137

[B10] Nolten WE Rueckert PA Elevated free cortisol index in pregnancy: possible regulatory mechanisms American journal of obstetrics and gynecology 1981 139 492 498 6258437 10.1016/0002-9378(81)90331-8

[B11] Neaves WB Billingham RE Transplantation of the testis Transplantation 1979 28 163 165 10.1097/00007890-197909000-00001 115123

[B12] Benediktsson R Calder AA Edwards CR Seckl JR Placental 11 beta-hydroxysteroid dehydrogenase: a key regulator of fetal glucocorticoid exposure Clinical endocrinology 1997 46 161 166 10.1046/j.1365-2265.1997.1230939.x 9135697

[B13] Stewart PM Rogerson FM Mason JI Type 2 11 beta-hydroxysteroid dehydrogenase messenger ribonucleic acid and activity in human placenta and fetal membranes: its relationship to birth weight and putative role in fetal adrenal steroidogenesis The Journal of clinical endocrinology and metabolism 1995 80 885 890 10.1210/jc.80.3.885 7883847

[B14] McTernan CL Draper N Nicholson H Chalder SM Driver P Hewison M Kilby MD Stewart PM Reduced placental 11beta-hydroxysteroid dehydrogenase type 2 mRNA levels in human pregnancies complicated by intrauterine growth restriction: an analysis of possible mechanisms The Journal of clinical endocrinology and metabolism 2001 86 4979 4983 10.1210/jc.86.10.4979 11600574

[B15] Wyrwoll CS Seckl JR Holmes MC Altered placental function of 11beta-hydroxysteroid dehydrogenase 2 knockout mice Endocrinology 2009 150 1287 1293 18845627 10.1210/en.2008-1100 PMC6435187

[B16] Langley SC Jackson AA Increased systolic blood pressure in adult rats induced by fetal exposure to maternal low protein diets Clinical science 1994 86 217 222; discussion 121 8143432 10.1042/cs0860217

[B17] Aufdenblatten M Baumann M Raio L Dick B Frey BM Schneider H Surbek D Hocher B Mohaupt MG Prematurity is related to high placental cortisol in preeclampsia Pediatric research 2009 65 198 202 10.1203/PDR.0b013e31818d6c24 19047954

[B18] Fowden AL Forhead AJ Coan PM Burton GJ The placenta and intrauterine programming Journal of neuroendocrinology 2008 20 439 450 10.1111/j.1365-2826.2008.01663.x 18266944

[B19] Langley-Evans SC Phillips GJ Benediktsson R Gardner DS Edwards CR Jackson AA Seckl JR Protein intake in pregnancy, placental glucocorticoid metabolism and the programming of hypertension in the rat Placenta 1996 17 169 172 10.1016/S0143-4004(96)80010-5 8730887

[B20] Bertram C Trowern AR Copin N Jackson AA Whorwood CB The maternal diet during pregnancy programs altered expression of the glucocorticoid receptor and type 2 11beta-hydroxysteroid dehydrogenase: potential molecular mechanisms underlying the programming of hypertension in utero Endocrinology 2001 142 2841 2853 10.1210/en.142.7.2841 11416003

[B21] Lesage J Blondeau B Grino M Breant B Dupouy JP Maternal undernutrition during late gestation induces fetal overexposure to glucocorticoids and intrauterine growth retardation, and disturbs the hypothalamo-pituitary adrenal axis in the newborn rat Endocrinology 2001 142 1692 1702 10.1210/en.142.5.1692 11316731

[B22] Hahn T Barth S Graf R Engelmann M Beslagic D Reul JM Holsboer F Dohr G Desoye G Placental glucose transporter expression is regulated by glucocorticoids The Journal of clinical endocrinology and metabolism 1999 84 1445 1452 10.1210/jc.84.4.1445 10199793

[B23] Fowden AL Ward JW Wooding FP Forhead AJ Constancia M Programming placental nutrient transport capacity The Journal of physiology 2006 572 5 15 16439433 10.1113/jphysiol.2005.104141 PMC1779642

[B24] Audette MC Greenwood SL Sibley CP Jones CJ Challis JR Matthews SG Jones RL Dexamethasone stimulates placental system A transport and trophoblast differentiation in term villous explants Placenta 2010 31 97 105 10.1016/j.placenta.2009.11.016 20045184

[B25] Jones HN Ashworth CJ Page KR McArdle HJ Cortisol stimulates system A amino acid transport and SNAT2 expression in a human placental cell line (BeWo) American journal of physiology Endocrinology and metabolism 2006 291 E596 603 10.1152/ajpendo.00359.2005 16621896

[B26] Mackenzie B Erickson JD Sodium-coupled neutral amino acid (System N/A) transporters of the SLC38 gene family Pflugers Archiv: European journal of physiology 2004 447 784 795 10.1007/s00424-003-1117-9 12845534

[B27] Murthi P Fitzpatrick E Borg AJ Donath S Brennecke SP Kalionis B GAPDH, 18 S rRNA and YWHAZ are suitable endogenous reference genes for relative gene expression studies in placental tissues from human idiopathic fetal growth restriction Placenta 2008 29 798 801 10.1016/j.placenta.2008.06.007 18684503

[B28] Fowden AL Sferruzzi-Perri AN Coan PM Constancia M Burton GJ Placental efficiency and adaptation: endocrine regulation The Journal of physiology 2009 587 3459 3472 10.1113/jphysiol.2009.173013 19451204 PMC2742275

[B29] Boileau P Mrejen C Girard J Hauguel-de Mouzon S Overexpression of GLUT3 placental glucose transporter in diabetic rats The Journal of clinical investigation 1995 96 309 317 10.1172/JCI118036 7615800 PMC185202

[B30] Lesage J Hahn D Leonhardt M Blondeau B Breant B Dupouy JP Maternal undernutrition during late gestation-induced intrauterine growth restriction in the rat is associated with impaired placental GLUT3 expression, but does not correlate with endogenous corticosterone levels J Endocrinol 2002 174 37 43 10.1677/joe.0.1740037 12098661

[B31] Shin BC Fujikura K Suzuki T Tanaka S Takata K Glucose transporter GLUT3 in the rat placental barrier: a possible machinery for the transplacental transfer of glucose Endocrinology 1997 138 3997 4004 10.1210/en.138.9.3997 9275091

[B32] Takata K Kasahara T Kasahara M Ezaki O Hirano H Ultracytochemical localization of the erythrocyte/HepG2-type glucose transporter (GLUT1) in cells of the blood-retinal barrier in the rat Investigative ophthalmology & visual science 1992 33 377 383 1740368

[B33] Gould GW Holman GD The glucose transporter family: structure, function and tissue-specific expression The Biochemical journal 1993 295 Pt 2 329 341 8240230 10.1042/bj2950329 PMC1134886

[B34] Das UG He J Ehrhardt RA Hay WW Jr Devaskar SU Time-dependent physiological regulation of ovine placental GLUT-3 glucose transporter protein American journal of physiology Regulatory, integrative and comparative physiology 2000 279 R2252 2261 11080093 10.1152/ajpregu.2000.279.6.R2252

[B35] Jansson N Pettersson J Haafiz A Ericsson A Palmberg I Tranberg M Ganapathy V Powell TL Jansson T Down-regulation of placental transport of amino acids precedes the development of intrauterine growth restriction in rats fed a low protein diet The Journal of physiology 2006 576 935 946 16916910 10.1113/jphysiol.2006.116509 PMC1892642

